# Real-Time and Efficient Traffic Information Acquisition via Pavement Vibration IoT Monitoring System

**DOI:** 10.3390/s21082679

**Published:** 2021-04-10

**Authors:** Zhoujing Ye, Guannan Yan, Ya Wei, Bin Zhou, Ning Li, Shihui Shen, Linbing Wang

**Affiliations:** 1 National Center for Materials Service Safety, University of Science and Technology Beijing, Haidian District, Beijing 100083, China; yezhoujing@ustb.edu.cn (Z.Y.); b20150406@xs.ustb.edu.cn (G.Y.); 2 Department of Civil Engineering, Tsinghua University, Haidian District, Beijing 100084, China; yawei@mail.tsinghua.edu.cn; 3 Yunnan Research Institute of Highway Science and Technology, Panlong District, Kunming 650051, China; zhoubin236@126.com (B.Z.); lnbb1010@163.com (N.L.); 4 Rail Transportation Engineering, Penn State University, Altoona, PA 16601, USA; szs20@psu.edu; 5 Virginia Tech, Blacksburg, VA 24061, USA

**Keywords:** road-embedded system, pavement vibration, internet of things, traffic information, real-time monitoring

## Abstract

Traditional road-embedded monitoring systems for traffic monitoring have the disadvantages of a short life, high energy consumption and data redundancy, resulting in insufficient durability and high cost. In order to improve the durability and efficiency of the road-embedded monitoring system, a pavement vibration monitoring system is developed based on the Internet of things (IoT). The system includes multi-acceleration sensing nodes, a gateway, and a cloud platform. The key design principles and technologies of each part of the system are proposed, which provides valuable experience for the application of IoT monitoring technology in road infrastructures. Characterized by low power consumption, distributed computing, and high extensibility properties, the pavement vibration IoT monitoring system can realize the monitoring, transmission, and analysis of pavement vibration signal, and acquires the real-time traffic information. This road-embedded system improves the intellectual capacity of road infrastructure and is conducive to the construction of a new generation of smart roads.

## 1. Introduction

Roadway transportation has the characteristics of high density, wide distribution, and great flexibility, which can satisfy people’s basic needs of transportation. However, traffic jams, traffic accidents, and road damage have become increasingly prominent with the increase of travel demand [1,2,3]. It is necessary to monitor the count, speed, classification, and weight of vehicles to promote transportation efficiency. Various systems for traffic monitoring have been developed in recent decades, which can be divided into the non-embedded type and the embedded type.

Non-embedded monitoring systems include camera, radar, ultrasonic wave, and laser, which are easy to install and cause no damage to the pavement structure [4,5]. However, they are prone to human destruction and severe weather, and the monitoring information and precision is limited. GPS and mobile communication equipment installed in the car can also acquire traffic information [6], but not all vehicles can be covered due to the high cost and the privacy concerns. In addition, the remote sensing technology is used to capture the traffic information on the two-dimensional plane instead of single-point traffic monitoring [7]. However, such technology demands a vast amount of investment capital and has low accuracy in single vehicle monitoring.

For embedded monitoring, the Weigh-in-Motion (WIM) system is used widely to monitor traffic information, including high-speed WIM and low-speed WIM [8,9]. High-speed WIM, often installed at main lanes, can acquire the information of vehicle type, speed, and weight. It is mainly used for traffic flow control and overload warning. The error of high-speed WIM ranges from 10–25%. The sensors used mainly include loop sensors, piezoelectric sensors, and fiber optic sensors. The low-speed WIM, often installed at toll stations, is mainly used to detect the overloading of vehicles and to determine an overweight penalty. The vehicle speed is controlled within 5–15 km/h, and the error is controlled in the range of 3–5%. The sensors used consist of strain gauge sensors and quartz piezoelectric sensors. The sensor technology is a key technology in WIM. Commonly used sensors include stress and strain sensors, piezoelectric sensors, fiber optic sensors, as well as vibration sensors.

Regarding stress and strain sensors, Zhang et al. [10] obtained the vehicle wheel base and number of axles through measuring pavement dynamic strain under vehicle load, and then made a classification for vehicles by support vector machine to extract classification characteristics from strain response. Xue et al. [11] collected the stress and strain signals under vehicle load by employing the embedded asphalt strain sensor and pressure sensor, and performed an inverse computation on vehicle axle load, wheel base, and traffic flow information. For piezoelectric sensors, Mazurek et al. [12] produced the piezoelectric sensor with Polyvinylidene Fluoride (PVDF) and performed a dynamic weighting experiment, proving the good performance of the piezoelectric sensor in WIM. Zhang et al. [13] constructed a mathematical model between the voltage output of cement-based piezoelectric sensors and the traffic flow, realizing traffic flow, vehicle speed, and dynamic weighting. Xu et al. [14] applied piezoelectric ceramic (PZT) materials to prepare the cement-based piezoelectric sensor, and established a set of piezoelectric sensor systems consisting of a prepared piezoelectric sensor, an amplifier, a multi-channel data acquisition instrument, and monitoring software. In terms of fiber optic sensors, Zhao et al. [15] developed a vehicle classification prototype system by using the distributed fiber optic vibration sensing technology, and presented a vehicle classification method to obtain the axle pattern, vehicle speed, and frequency domain characteristics of different types of vehicles. Dong et al. [16] deployed fiber grating sensors in airport asphalt pavement for monitoring the pavement dynamic response under aircraft load, and aircraft load deviation position, speed, dynamic response duration and other information were obtained through collecting and analyzing field monitoring data. For Integrated Electronics Piezo-Electric (IEPE) accelerometers, Hostettler [17] adopted the IEPE accelerometer to collect pavement vibration signals under moving vehicle load, analyzed the time-frequency domain characteristics of measured signals, and calculated the number of axles. Levenberg [18] installed the high precision single-axle IEPE accelerometer (KB12VD) on roads to record the pavement vertical vibration signals of passing vehicles, and then calculate vehicle speed and axle load distribution.

However, due to the technical constraints, traditional stress and strain sensors, piezoelectric sensors, and IEPE accelerometers, should be equipped with the data collection device when being applied in roads with poor durability and insufficient data processing and communication functions. Such a data collection device powered by the external power supply has high energy consumption and can hardly have real-time processing with the large amount of data collected.

With the development of the Internet of Things (IoT) technology, micro-electro-mechanical systems (MEMS) vibration sensors exhibit technical advantages in low power consumption, miniaturization, and extensibility, and realize wireless communication and edge computing by virtue of chip integration technology and wireless communication technology [19,20]. Bajwa et al. [21,22,23] advanced a wireless sensor network used for vehicle classification, including the acceleration sensor monitoring pavement vibration, the magnetic induction sensor monitoring vehicle arrival and departure, and data access equipment to realize vehicle classification and axle load estimation. Ma et al. [24] employed a wireless acceleration sensor and a magnetic induction sensor in vehicle classification and counting, where the former was used for detecting the number of vehicles, and the latter was used for monitoring vehicle arrival and departure and vehicle speed estimation. Stocker et al. [25] applied the CEF C3M01 acceleration sensor produced by Webrosensor Oy in measuring vehicle-caused pavement vibration, and used the machine learning algorithm in vehicle classification and counting. Kleyko et al. [26] deployed the magnetic induction sensor and acceleration sensor on the roadside to process the monitoring data using the feature-free data smashing approach, aiming to accomplish vehicle classification and traffic flow counting. Huang et al. [27] adopted the wireless MEMS acceleration sensor to measure pavement vibration under moving vehicle load. Traffic counting and vehicle classification were made by eradicating the noise in vibration signals with the proposed algorithm. In the above studies, the data collected by the sensors were mostly processed offline locally, and some of the sensors were deployed on the roadside. The wireless vibration sensors embedded in pavement often fail not long after installation due to the cyclic vehicle loading and insufficient power supply.

In order to improve the durability and efficiency of the road-embedded monitoring system, the Internet of Things (IoT) technology is used to develop a pavement vibration monitoring system in this study. IoT is the network of things, with clear element identification, embedded with software intelligence, sensors, and ubiquitous connectivity to the Internet [28,29,30]. The IoT system can access different types of sensors and actuators, can be compatible with a variety of communication protocols, and can realize distributed computing integrated with artificial intelligence (AI) technology [31,32,33,34,35]. The developed pavement vibration IoT monitoring system has significant advantages in real-time monitoring, intelligent computation, and system scalability. Table 1 shows the advantages of the pavement vibration IoT monitoring system compared with commercial traffic monitoring systems.

## 2. System Composition

**Figure 1:**
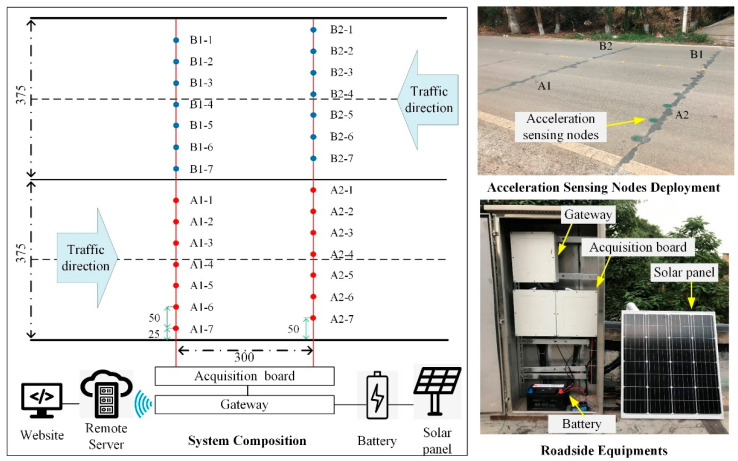
Pavement vibration IoT monitoring system.

The pavement vibration IoT monitoring system in this study consists of acceleration sensing nodes, a gateway, and a cloud platform, which has been previously applied in the field, realizing the real-time, efficient, possibly long-term monitoring of traffic information, as shown in Figure 1.

The system was deployed on 320 National Highway in Kunming, Yunnan Province. Two columns of sensing nodes were deployed crosswise in each lane, and each column had seven sensing nodes. The distance between adjacent nodes was 50 cm. The acceleration sensing nodes collected pavement vibration signals at the sampling frequency of 500 Hz. The data of all sensing nodes were gathered to the acquisition board, which was further connected to the gateway. The gateway communicated with the remote server via the 4G wireless network. This system is powered by 12 V battery and solar energy and visual monitoring data on the website.

## 3. Acceleration Sensing Node

Acceleration sensing node, as the front-end sensing device of the system, is used for collecting the pavement vibration induced by the moving vehicle load. The acceleration sensing node adopts the MEMS technology, which supports the integration of MEMS sensors, processors, and other electronic components, and thus realizes low-power, high-integration, and intelligent monitoring.

### 3.1. Packaging Design

**Figure 2:**
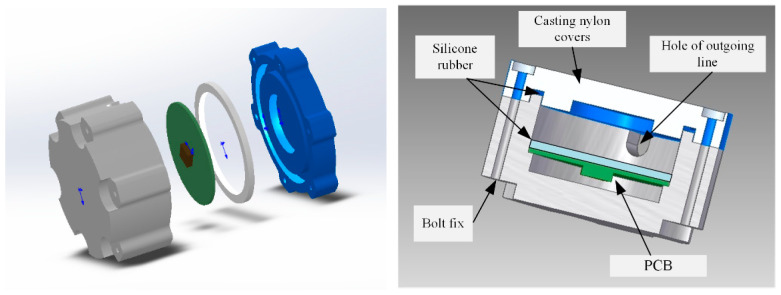
Composition of acceleration sensing node.

The acceleration sensing node consists of casting nylon covers, a waterproof rubber gasket, and a Printed Circuit Board (PCB), as shown in Figure 2.

The dimensions of the acceleration sensing node may be designed according to the practical engineering requirements. Given that the diameter of the drilling hole is 10 cm, the node was specifically designed as a cylinder with the outer diameter of 8 cm and the height of 5 cm, to match with the drilling process. Casting nylon packaging can provide the sensing node with better compressive load bearing capacity. In order to improve the waterproof performance of the node, the silicone rubber is filled to cover the PCB. This prevents the water from infiltrating into the package box and causing any damage to the PCB under hydrodynamic pressure of pavement. Note that the silicone rubber should not fill in the entire cavity in casting, otherwise the PCB will be damaged by forces when the silicone rubber is subject to the outer wall after thermal expansion in practical applications. To ensure long-term monitoring, the sensing node uses wired communication and power supply. The hole of the outgoing line is arranged on the side wall of the acceleration sensing node, so that it will not directly suffer from the vehicle load.

### 3.2. Printed Circuit Board

**Figure 3:**
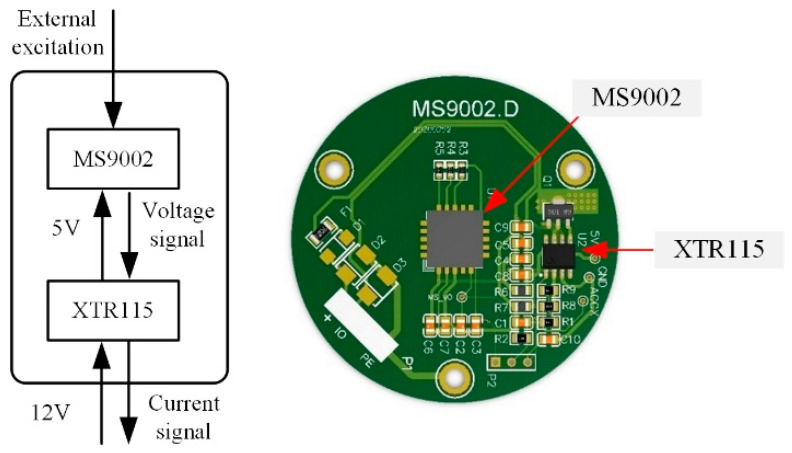
The Printed Circuit Board (PCB) of the acceleration sensing node.

The PCB achieves the electrical interconnection among all components, which is the core part to realize the data acquisition of acceleration sensing node, as shown in Figure 3.

PCB consists of an accelerometer (MS9002), a current loop transmitter (XTR115), and capacitors and resistances. The deployment of PCB is centered around the accelerometer, and all electronic components are evenly and compactly allocated around. The PCB can convert the external mechanical vibration to the analog current signal output through the two main components, the accelerometer and current loop transmitter. The accelerometer outputs vary voltage signals in response to the stimulus of vibration in the external environment, and the voltage is in a linear relationship with vibration. The voltage signal is converted into the current signal of 4–20 mA by using the current loop transmitter. Simultaneously, the current loop transmitter lowers the voltage from 12 V to the 5 V to power the accelerometer.

In field monitoring, multi-node real-time monitoring usually is adopted. For its large data communication, long-distance transmission, and high environmental noise, if the voltage signal of the accelerometer is output directly, the voltage signal will be vulnerable to the interference of environmental noise in transmission. Moreover, considering the long-distance transmission, the resistance along the transmission line may lead to the voltage drop. Thus, for mitigating the influence of environmental noise and voltage drop, the PCB carries the XTR115 which can convert the voltage signal to the current signal output. As the current has strong anti-noise capability in transmission and no voltage drop is involved, the aforementioned problems can be well solved. Besides, when analog signal outputs are used, free from the influence of digital communication bandwidth, it is able to meet the requirements in mass data real-time transmission at multi-nodes monitoring.

### 3.3. Circuit Design

**Figure 4:**
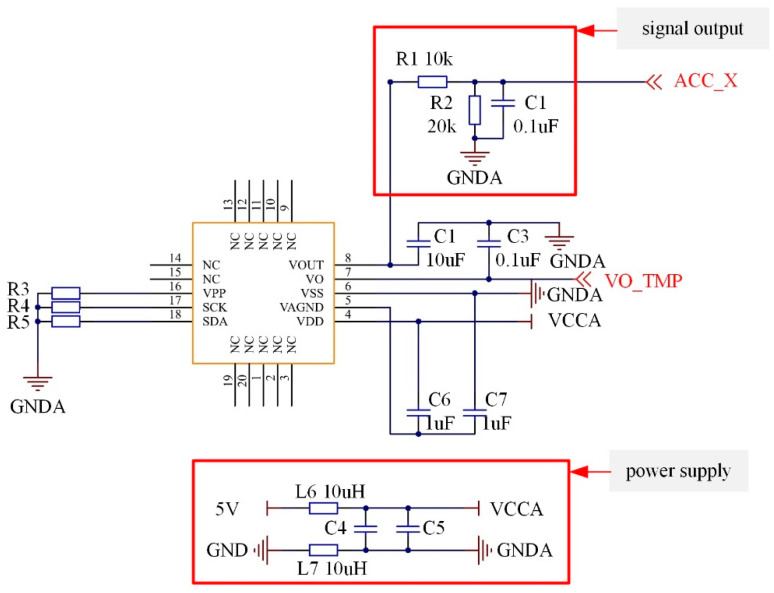
Circuit diagram of the accelerometer.

To exert the efficacy of all components and ensure system stability, the circuit design of PCB should not only take into account the connection layout of electronic components, but also examine many other factors such as noise reduction, electromagnetic protection, and heat dissipation. Figure 4 shows the circuit diagram of the accelerometer.

A first-order filter circuit consisting of 10 kΩ resistance and 0.1 μF capacitance is set at the accelerometer signal output end (ACC_X), which can filter the high-frequency noise. Additionally, the 20 kΩ resistor is used as the voltage dividers, and the output voltage of the accelerometer is controlled within 0–3.3 V to match with the input voltage range of XTR115 and the Analog-to-Digital Converter. The power end of the accelerometer (5 V and GND) sets the L6 and L7 inductance to physically isolate the external 5 V power supply, stabilize the current, and reduce the high-frequency interference from the external power supply. C4 and C5 capacitance function in voltage stabilization.

**Figure 5:**
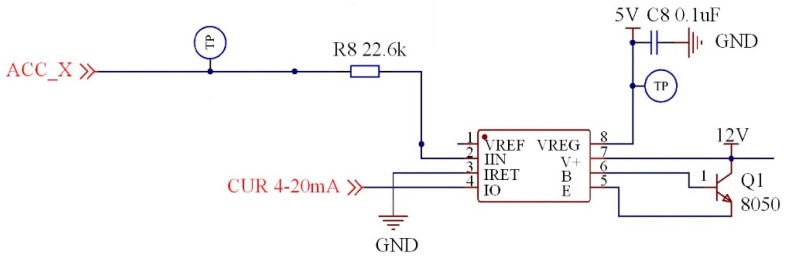
Circuit diagram of the XTR115.

Figure 5 shows the circuit diagram of the XTR115. The Negative-Positive-Negative (NPN) triode Q1 is used as the current amplifier. The magnified current signal is input via Pin 5. The energy of magnified current signal is powered by 12 V power supply. On the other side, the voltage signal of the accelerometer is converted to the current signal via R8. The variable current is input through Pins 2 and 3, which can be taken as the command for the magnified current control. Through the modulation of XTR115 chip, Pin 4 outputs the 4–20 mA current signal that can reflect the pavement vibration, and the XTR115 chip lowers the voltage from 12 V to 5 V to power the accelerometer.

## 4. Gateway

### 4.1. Acquisition Board

**Figure 6:**
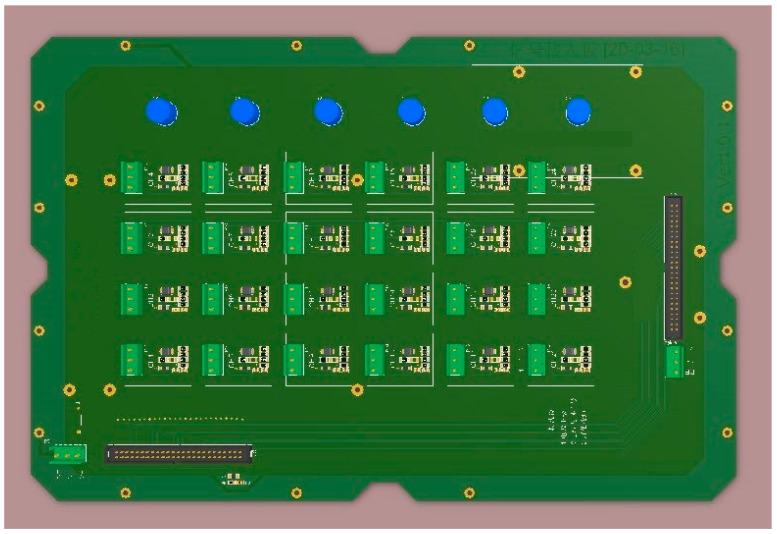
The acquisition board.

The acquisition board can be connected with 24 acceleration sensing nodes, which are powered by 12 V voltage, as shown in Figure 6.

**Figure 7:**
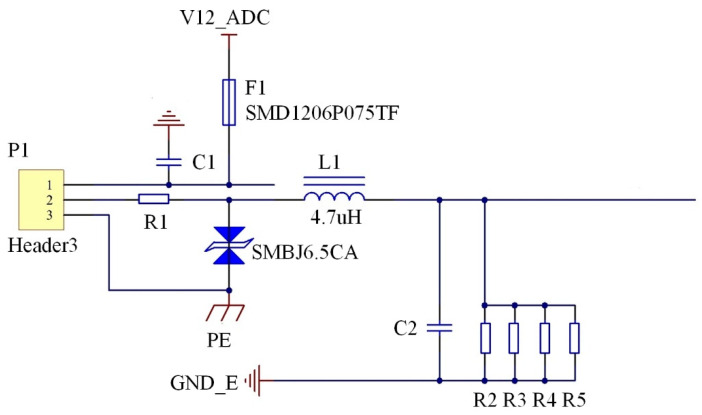
Circuit diagram of acquisition board.

The current signal output via multiple nodes on the acquisition board is converted into the voltage signal, as shown in Figure 7.

The current signal of the sensing node is input via the V12_ADC and output via GND_E. The self-recovery fuse (SMD1206P075TF) in the pathway has the dual functions of overcurrent overheating protection and automatic recovery. The resistance (R1) and transient voltage suppressor (SMBJ6.5CA) are set to prevent the circuit component from being damaged by high transient energy, and to avoid electrical surges and short circuits. Moreover, the inductance (L1) is set to pass the direct current and obstruct the alternating current. The four resistors, namely R2-R5, convert the current into the voltage. The multiple resistors in parallel can effectively dissipate the heat so as to prevent resistance change caused by excessively high temperature. This can stabilize voltage and improve the accuracy of the conversion from the current to the voltage, and the capacitor (C2) further stables the voltage.

### 4.2. Gateway Composition

**Figure 8:**
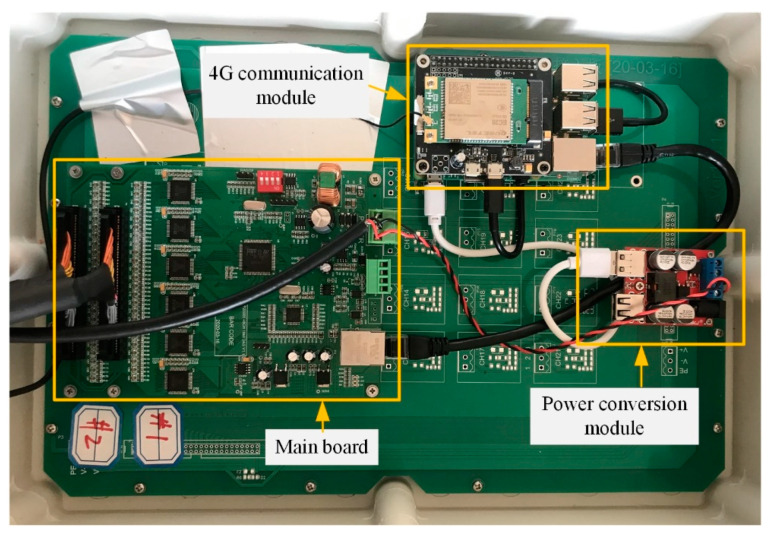
Gateway composition.

The gateway realizes the communication between the sensor network and the remote server, which consists of a main board, a 4G communication module, and a power conversion module, as shown in Figure 8.

The main board is equipped with six Analog-to-Digital Converters (AD7606) to convert the voltage signal transmitted by the acquisition board into the digital signal, equipped with CPU (STM32F103VET6) to store and process the digital data, and also equipped with an Ethernet controller (W5500) to transmit the data to the 4G communication module via the cable. In this way, the data can be sent to the remote server wirelessly. In addition, the gateway is powered by the 12 V lithium-ion battery charged by solar energy, which ensures the sufficient power supply of the battery. Through the power conversion module, the power supply is reduced from 12 V to 5 V in voltage, which can be used to power the 4G communication module.

### 4.3. Communication Mechanism

To improve the efficiency of wireless transmission and decrease the communication energy consumption and cost, data compression technology and overlapping–uploading mechanisms are used.

(1) Data compression technology

The gateway uses the Zlib compression algorithm to reduce the amount of data uploaded. Table 2 shows the compression ratio of Zlib.

The Zlib compression algorithm compresses the data through seeking repetitive strings in the input data. For the data packages in a fixed size, the compression rate is within 31–33%. System energy consumption and communication cost can be efficiently reduced by uploading after compression.

(2) Overlapping upload mechanism

**Figure 9:**
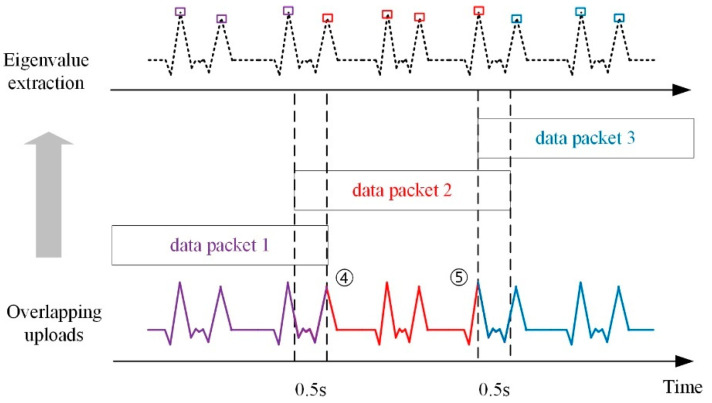
The overlapping upload mechanism.

In order to prevent the loss of characteristic data, the overlapping uploading mechanism is adopted, meaning that the adjacent data packages uploaded by the gateway are allowed for 0.5 s repetition, as shown in Figure 9.

The uploaded packages are uniform in format and length. In field monitoring, feature data may exactly fall at the end or beginning of the data packages, such as the fourth peak and the fifth peak in Figure 9. This will lead to the missing of peak data in data package 1 and data package 3. Through the overlapping uploading mechanism, it is almost guaranteed that the decompressed original data will not lose the feature data after being uploaded. For instance, the missing peak data in data package 1 can be recognized in data package 2. Similarly, the fifth peak can be also identified in data package 2. As the vibration data correspond to the unique ID of time, repetitively recognized feature data can be merged according to the same time ID.

## 5. Cloud Platform

### 5.1. Remote Server

**Figure 10:**
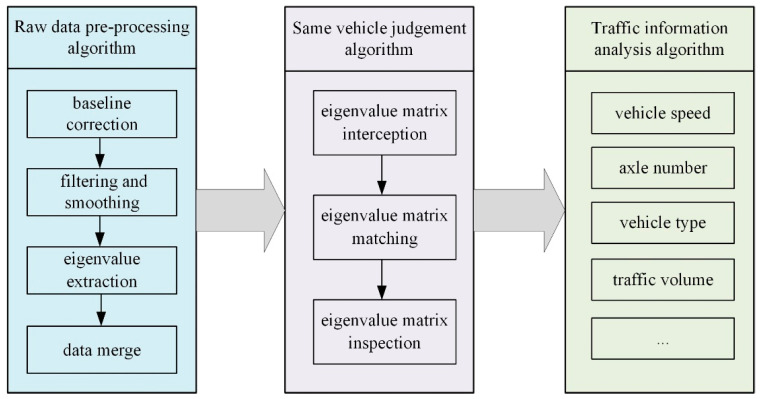
Data processing algorithms in remote server.

The remote server decompresses the uploaded data packages and analyzes raw vibration data by using the data processing algorithms, including the raw data pre-processing algorithm, same vehicle judgement algorithm, and traffic information analysis algorithm, as shown in Figure 10.

(1) Raw data pre-processing

The acceleration sensing nodes generate temperature drift under the influence of temperature, causing the difference in the reference value of pavement vibration data monitored at different nodes. Therefore, targeting at the monitoring data uploaded by every sensing node, the least square method is employed to derive the reference value of data in this section. The pavement vibration data at each node with zero as the reference value can be obtained by subtracting the raw vibration data from the obtained reference value. Subsequently, filtering and smoothing processing is performed on raw pavement vibration data to extract the peak and corresponding time information [36].

**Figure 11:**
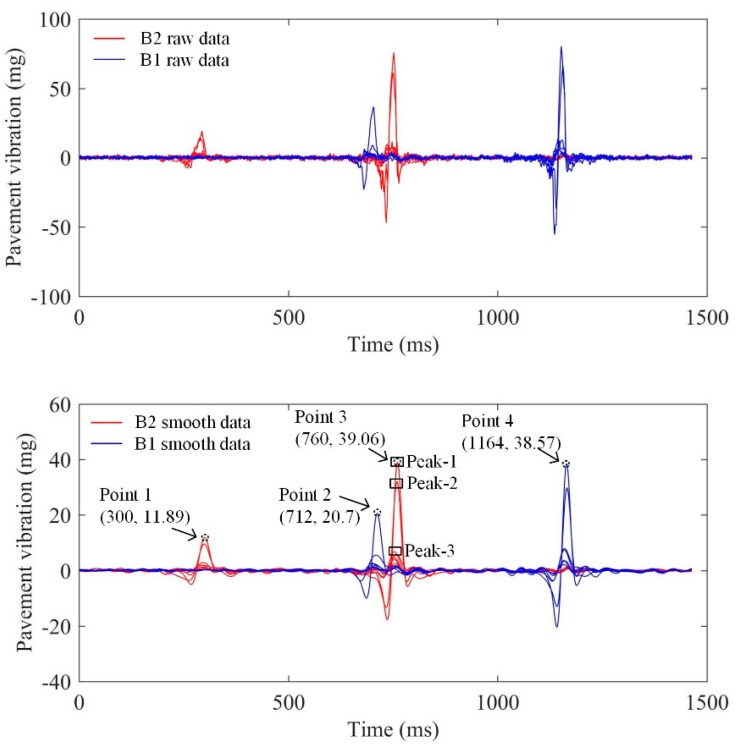
Pavement vibration data of a two-axle vehicle passing through the monitoring area.

Figure 11 shows the pavement vibration data of a two-axle vehicle passing through the monitoring area. When the vehicle passes through the monitoring area, the left and right wheels on the same axle do not press the sensing nodes in the same column at the same time, leading to the time difference in the resulting peak. If the peak time difference at sensing nodes is less than 10 ms, it can be judged that the peak values are generated from the same axle at the same time. As shown in Figure 11, the peaks (Peak-1, Peak-2, and Peak-3) can be considered to be generated under the action of the same axle, and the time that the axle corresponds to takes the time at Peak-1 with the maximum peak value as the reference value, aiming to obtain the pavement vibration characteristic data (Point 1, Point 2, Point 3, and Point 4) under the action of the same axle.

(2) Same vehicle judgement

The characteristic data of each axle (peak and corresponding time) can be obtained through performing the analysis with raw data pre-processing algorithms. However, it remains unknown which axles belong to the same vehicle. Thus, it is of necessity to make the same vehicle judgment. The same vehicle judgment includes three steps.

The first step is to judge whether the characteristic data of nodes in the same column correspond to the same vehicle, and then form a characteristic matrix of each vehicle based on the nodes in the same column. The second step is to match the characteristic matrix of two columns of nodes to judge whether the characteristic matrix formed at different columns of nodes corresponds to the same vehicle. The last step is to verify previous judgments of the vehicle matrix in accordance with the characteristics of the vehicle rear axle, such as single-axis double-tire, tandem axle, and tridem axle.

(3) Traffic information analysis

Based on the eigenvalue matrix of each vehicle, analysis algorithms such as vehicle speed, distance between axles, number of axles, location of vehicle load, driving direction, traffic volume, and vehicle type are developed to acquire the comprehensive information of the vehicle [37].

① Speed and wheelbase

Vehicle speed can be calculated through the peak time difference between two columns of nodes in the same axle and the spacing between two columns of nodes. With the known vehicle speed, the wheelbase of adjacent axles can be calculated based on the peak time difference under the action of the adjoining axle.

② Number of axles and vehicle type

The number of rows in the vehicle characteristic matrix corresponds to the number of axles of the vehicle. Vehicle classification can be made in line with relevant regulations through inputting vehicle speed, average wheelbase, total amplitude, load effect position, number of axles, and maximum frequency into the artificial neural network model.

③ Location of the vehicle load

Nodes at load effect position will generate obvious peaks, while the vibration amplitude at nodes deviating from the load effect position will dramatically decline. When the vehicle passes through the monitoring area, the node which generates the maximum amplitude can be considered the location of the vehicle load.

④ Driving direction

The monitoring area has deployed two columns of nodes. If the time of the amplitude generated by the first column of nodes precedes that of the second column, then the vehicle drives from the first column to the second column. If the contrary happens, the vehicle drives in the reverse direction.

⑤ Traffic volume

Based on above monitoring results, the number of vehicles and corresponding vehicle models in a period of time can be obtained to determine the traffic volume of different vehicles in this road section.

By using the algorithms mentioned above, traffic information can be extracted from the vibration data, and the database MongoDB is used for traffic information storage and calling. The monitoring accuracy of the system for traffic information is greater than 90%, which satisfies the practical application needs. Errors may occur when the vehicles pass the section at a low speed (less than 5 km/h) or press the spikes.

### 5.2. Website Interface

**Figure 12:**
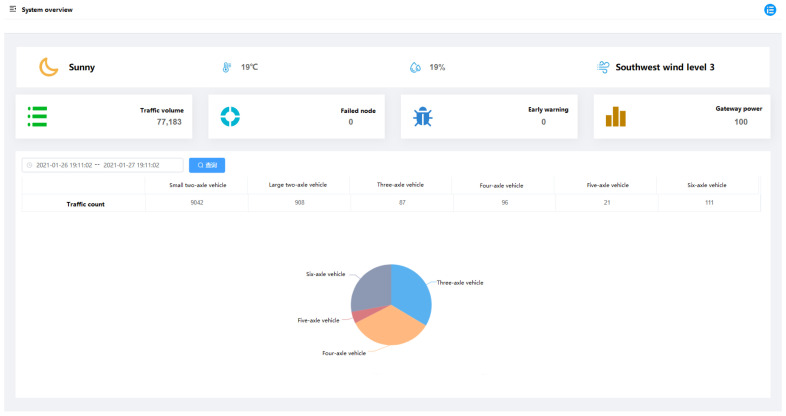
Website interface.

The website interface provides a platform for the interaction between the user and the system. The user acquires the data resources by visiting the website. In addition, the user can input the verified data of vehicle weight and type into the database as the calibration data via the website. The website interface includes system overview, equipment management, data management, and project information, as shown in Figure 12.

The website displays the environment information, traffic information, warning information, and equipment information captured by the monitoring system. The monitoring data can be exported and inquired, including the traffic count and detailed information of large vehicles.

**Figure 13:**
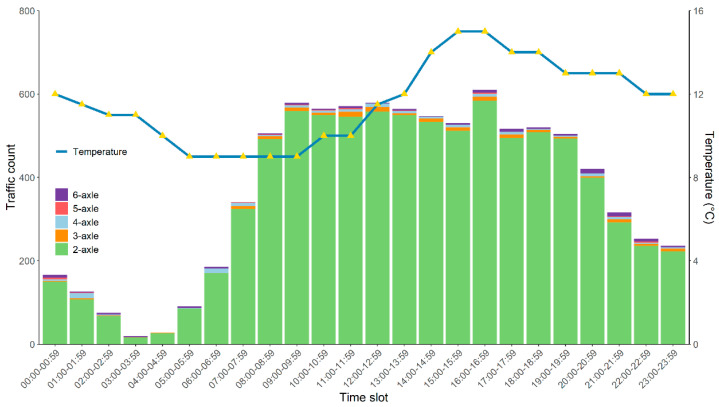
Variation in traffic count and temperature over hours.

Figure 13 shows the variation in traffic count and temperature over time in one day. The traffic volume reaches the minimum in 3:00–4:00, with merely 20 vehicles per hour. Then it gradually increases, and sharply increases after 7:00. The traffic volume remains at nearly 600 vehicles per hour until 9:00, and the momentum continues until 20:00. After 20:00, the traffic volume slowly decreases. During the period of 9:00–20:00, the temperature gradually increases from 9 °C at 9:00 to 15 °C at 17:00, and then slowly decreases to 13 °C until 20:00. When the traffic volume is large, the temperature rises and remains relatively high. The minimum temperature of 9 °C occurs at 5:00–9:00. During this period, the traffic volume is relatively small.

The system can acquire the detailed information of vehicles with over three axles, including vehicle lane, time, number of axles, vehicle speed, axle length, and vibration amplitude, as shown in Table 3.

In Table 3, the range of monitored speed is 25 km/h to 45 km/h. The number of vehicle axles ranges between three and six. As the maximal number of vehicle axles is six, the axial length information includes the total axle length, distance between axle 1 and axle 2, distance between axle 2 and axle 3, distance between axle 3 and axle 4, distance between axle 4 and axle 5, and distance between axle 5 and axle 6. The distance between axles varies from different types of vehicle. Even for the same type of vehicle, the distance between axles is also different. The classification for vehicle type can be made according to the axle length of vehicles and the local classification standards. Vibration amplitude includes the total amplitude of all nodes in the lane, and also the total amplitude of front-column nodes (A1 and B2) and rear-column nodes (A2 and B1). Thus, it can be seen that vehicles with a larger number of axles may not have a greater total amplitude, and pavement vibration amplitude is subject to multiple factors.

## 6. Conclusions

The pavement vibration IoT monitoring system consists of multi-acceleration sensing nodes, a gateway, and a cloud platform, which enables the monitoring, transmission, and analysis of pavement vibration signal, and acquires real-time traffic information. The conclusion is as follows:

(1) The acceleration sensing node replaces voltage signal by the current signal to reduce noise interference in the signal transmission process and voltage drop caused by long-distance transmission. Different from the wireless transmission, the installation of wired sensing nodes requires pavement drilling and cutting, but the damages can be avoided through prefabrication technologies. Wired transmission is more stable in practice, which manifests itself in data communication and power supply. In addition, the acceleration sensing node based on the MEMS technology can integrate low-cost and low-power electronic components through the circuit design of PCB, which realizes data acquisition, filtering, processing, and transmission at the sensing terminal.
(2) The acquisition board collects the current signals of each acceleration sensing node and converts them into voltage signals. The gateway converts the voltage signal into a digital signal and adopts the data compression technology and overlapping uploading mechanism to ensure the efficiency and stability of wireless transmission. The Zlib compression method with a compression rate of 32% decreases the amount of data transmitted wirelessly and reduces energy consumption and communication costs. The adjacent data packages uploaded by the gateway are allowed for 0.5 s repetition, which prevents the loss of feature data in wireless communication.
(3) The cloud platform includes a remote server and a website interface. The remote server decompresses the uploaded data packages and analyzes raw vibration data through using data processing algorithms, including a raw data pre-processing algorithm, a same vehicle judgement algorithm, and a traffic information analysis algorithm. The website visualizes temperature, vehicle lane, time, number of axles, vehicle speed, axle length, and vibration amplitude. The information can be used to assess the impact of traffic load and environmental factors on the pavement.

The IoT monitoring system acquires traffic information through pavement vibration monitoring and analysis. It is more advanced in real-time monitoring, intelligent computation, and system scalability, which makes itself distinguished from the systems of cameras, geo-magnetism, and WIM. Through larger deployment and real-time traffic monitoring, the system can not only provide data for traffic management and road maintenance, but also for autonomous vehicles. In the future, the influence of vehicle speed, vehicle weight, and vehicle type on the time-frequency characteristics of pavement vibration needs to be studied. In the meantime, it is necessary to evaluate the durability of the system to optimize and verify the data processing algorithm. It will be necessary to realize the intelligent interaction between infrastructure and X, such as LED lights, signs, and unmanned aerial vehicles (UAV), which can be included in the system as a broader effort to realize real-time and intelligent interactions.

## Figures and Tables

**Table 1:** Advantage of the developed pavement vibration Internet of Things (IoT) monitoring system.

Commercial Monitoring Method	System Type	Advantages of Our System
Video monitoring system	Non-embedded	Not affected by weather changes Compared with two-dimensional image data, vibration data can be processed more efficiently Dynamic response of pavement structure can be monitored
Geomagnetic monitoring system	Embedded	More monitoring parameters, not just traffic volume and speed High precision of single vehicle monitoring
Weigh-in-Motion system	Embedded	Low cost, about 1/3 of WIM Low energy, 12 V power supply Easy to install Good compatibility and scalability based on IoT

**Table 2:** The compression ratio of Zlib.

No.	Packet Size before Compression (Byte)	Packet Size after Compression (Byte)	Compression Ratio
1	1,488,020	488,529	32.83
2	1,488,020	473,625	31.83
3	1,488,020	472,366	31.74
4	1,488,020	478,555	32.16
5	1,488,020	490,769	32.98

**Table 3:** Detailed information of vehicles with over three axles.

Lane	Time	Number of Axles	Vehicle Speed (km/h)	Axle Length (m)	Vibration Amplitude (mg)
A	10/Jan/2021 23:16:03	3	44.26	[5.19, 3.86, 1.33, 0, 0, 0]	[1193.95, 820.05, 373.91]
A	10/Jan/2021 23:13:46	3	43.90	[4.66, 3.32, 1.34, 0, 0, 0]	[343.25, 73.06, 270.19]
B	10/Jan/2021 22:44:56	6	28.88	[12.31, 3.29, 1.35, 5.05, 1.31, 1.32]	[1108.05, 464.25, 643.80]
B	10/Jan/2021 22:41:40	4	38.85	[7.23, 1.88, 3.99, 1.36, 0, 0]	[276.28, 92.92, 183.36]
A	10/Jan/2021 22:31:26	6	28.57	[13.12, 3.30, 1.32, 5.88, 1.33, 1.29]	[1769.06, 831.29, 937.77]
A	10/Jan/2021 22:31:20	6	29.35	[12.79, 1.78, 2.72, 5.68, 1.30, 1.32]	[2224.27, 1247.13, 977.14]
B	10/Jan/2021 22:19:30	3	32.14	[5.73, 4.38, 1.36, 0, 0, 0]	[187.56, 82.31, 105.25]
A	10/Jan/2021 22:16:23	6	30.17	[12.95, 3.13, 1.36, 5.89, 1.29, 1.29]	[1909.21, 1428.77, 480.44]
A	10/Jan/2021 22:08:10	6	29.35	[13.63, 3.16, 1.40, 6.57, 1.35, 1.14]	[1283.00, 992.17, 290.83]
B	10/Jan/2021 22:03:32	6	25.12	[12.47, 3.29, 1.33, 5.52, 1.16, 1.17]	[331.62, 166.21, 165.41]

## Data Availability

Data is contained within the article.
